# MitoQ Loaded Chitosan-Hyaluronan Composite Membranes for Wound Healing

**DOI:** 10.3390/ma11040569

**Published:** 2018-04-07

**Authors:** Tamer M. Tamer, Maurice N. Collins, Katarina Valachová, Mohamed A. Hassan, Ahmed M. Omer, Mohamed S. Mohy-Eldin, Karol Švík, Rastislav Jurčík, Ľubomír Ondruška, Csaba Biró, Ahmad B. Albadarin, Ladislav Šoltés

**Affiliations:** 1 Polymer Materials Research Department, Advanced Technologies and New Materials Research Institute (ATNMRI), City of Scientific Research and Technological Applications (SRTA-City), New Borg El-Arab City, 21934 Alexandria, Egypt; ttamer85@gmail.com (T.M.T.); ahmedomer_81@yahoo.com (A.M.O.); mohyeldinmohamed@gmail.com (M.S.M.-E.); 2 Laboratory of Bioorganic Chemistry of Drugs, Institute of Experimental Pharmacology and Toxicology, Slovak Academy of Sciences, 84104 Bratislava, Slovakia; katarina.valachova@savba.sk (K.V.); ladislav.soltes@savba.sk (L.Š.); 3 School of Engineering, Bernal Institute, University of Limerick, V94 T9PX Limerick, Ireland; 4 Protein Research Department, Genetic Engineering and Biotechnology Research Institute (GEBRI), City of Scientific Research and Technological Applications (SRTA-City), New Borg El-Arab City, 21934 Alexandria, Egypt; m_adelmicro@yahoo.com; 5 Chemistry Department, Faculty of Science, University of Jeddah, Osfan, P. O. Box: 80203, 21589 Jeddah, Saudi Arabia; 6 Department of Toxicology and Laboratory Animals Breeding, Institute of Experimental Pharmacology and Toxicology, Slovak Academy of Sciences, 91954 Dobra Voda 360, Slovakia; karol.svik@savba.sk; 7 National Agricultural and Food Centre, Research Institute for Animal Production Nitra, Department of Small Farm Animals, 951 41 Lužianky, Slovakia; jurcik@vuzv.sk (R.J.); ondruska@vuzv.sk (Ľ.O.); 8 St. Elizabeth Cancer Institute Hospital, Department of Pathology, Bratislava, 84104, Slovakia; csaba.biro1675@gmail.com; 9 Department of Chemical Sciences, Bernal Institute, University of Limerick, V94 T9PX Limerick, Ireland; ahmad.b.albadarin@ul.ie

**Keywords:** mitochondrially-targeted antioxidant, skin wounds, hyaluronan, chitosan

## Abstract

Two self-associating biopolymers, namely chitosan (Ch) and a high-molar-mass hyaluronan (HA), were used to prepare membranes with the aim to protect and to enhance the healing of injured skin. A mitochondrially-targeted antioxidant—MitoQ—was incorporated into the mixture of biopolymers prior to their self-association. These three-component membranes were evaluated in detail utilising surface roughness measurements, contact angle measurements, hemocompatibility, and thrombogenicity analyses. Furthermore, in vivo application of Ch/HA/MitoQ membranes was assessed on injured rabbit and rat skin utilizing histological methods. The results showed that the prepared thrombogenic Ch/HA/MitoQ membranes had higher roughness, which allowed for greater surface area for tissue membrane interaction during the healing processes, and lower cytotoxicity levels than controls. MitoQ-loaded composite membranes displayed superior healing properties in these animal models compared to control membranes.

## 1. Introduction

Oxidative damage is associated with aging and a wide range of human disorders [1]. Production of reactive oxygen species (ROS), such as superoxide anion radical O_2_^●**–**^ and the oxidant H_2_O_2_, occurs during cellular respiration in mitochondrion. Healthy young cells are capable of detoxifying ROS using enzymes such as superoxide dismutases and catalase [2,3,4].

Damaged tissues, such as leg and decubitus ulcers or severe burns, often undergo acute and, subsequently, chronic inflammation. These tissues contain necrotic cells and cells in which oxygen uptake is insufficient due to damage of extracellular glycocalyx (state of hypoxia). It is well known that mitochondria in cells enhance the production of ROS, such as O_2_^●–^ and H_2_O_2_ forming ^●^OH radicals during hypoxia, and damaged cells are incapable of repair [5,6]. In general, it is known that ROS originate in elevated amounts from stressed cells. Therefore, it is favourable to limit the production of ROS in a site of chronic inflammation.

One way to effectively reduce an adverse flow of ROS from mitochondria is to use mitochondria targeted antioxidants (MTAs), which selectively enter mitochondria. Presently, MTAs are applied at the site of inflamed tissue at relatively low concentration for a prolonged period of time. The main requirement for the function of an antioxidant as a protective species for mitochondria is its capacity to penetrate through the cell-cytosolic-bilayer and to enter into the subcellular compartments, namely to the space between the inner and outer mitochondria membranes (lipid bilayers) and into the matrix of the mitochondrion [7].

In recent years, research has focused on the advancement of mitochondria-targeted antioxidants, such as MitoQ, the molecule which is composed of the coenzyme Q_10_ carried by the lipophilic triphenylphosphonium cation, often classified as the Skulatchev ion [8]. Due to its lipophilic tail, MitoQ migrates to the cell cytosolic membrane, diffuses through it easily, and accumulates in relatively high concentrations within mitochondria [9,10,11,12,13,14].

MitoQ exists in two forms: the oxidized form MitoQuinone and the reduced one MitoQuinol [15]. The drug active principle is related to coenzyme Q_10_, which serves as the donor-acceptor of electrons [16]. Since HA bears a negative charge and chitosan a positive one, the complexation of these two biopolymers results in the formation of stable composite membranes. Through setting an optimal ratio of Ch and HA, it is postulated that the rate of MitoQ release from the Ch/HA/MitoQ composite membranes may be controlled.

HA hydrogels are largely utilised in drug delivery, tissue engineering, and wound dressing applications, mainly due to their high water-retaining capacity, stability, biocompatibility, and tuneable viscoelastic properties [17]. During the inflammatory phase of wound healing endogenic high-molar-mass HA accumulates in the wound and functions as a regulator of early inflammation. The major functions of HA in this phase are the modulation of inflammatory cells and fibroblast cell migration, modulation of the synthesis of proinflammatory cytokines, and phagocytosis of invading microorganisms. In this phase, HA degradation products, such as low-molar-mass HA (2.5 × 10^5^ Da), can enhance early inflammation [18,19]. The HA fragments, which accumulate due to degradation of high-molar-mass HA, can initiate toll receptor-2 and toll receptor-4 induction of pro-inflammatory cytokines IL-6, TNF-α, and IL-1β. The proliferative phase overlaps with the remodelling phase, where keratinocytes differentiate to fibroblasts. During these phases the growth factors and cytokines released by the inflammatory cells induce fibroblast and keratinocyte migration and proliferation. In the second and third phases of healing endogenic HA binds with water, thereby contributing to a moist environment [20]. The levels of HA synthesized by both fibroblasts and keratinocytes are elevated during re-epithelisation where epithelial cells migrate across the new tissue to form a barrier between the wound and the environment [21]. As a result, HA is involved in the healing of acute wounds, accelerates healing of chronic skin damage, diabetic ulcers, and decubites [22,23,24,25]. HA has also been used as a coating to enhance cellular viability on synthetic tissue scaffolds [26].

Chitosan positively influences the healing process through faster formation of granular tissue in the initial stages of healing [27,28,29,30] with chitosan and its derivatives displaying antioxidative and antibiotic behaviour [31].

Although Ch/HA membranes have been used previously for wound healing [32], this paper focuses on the influence of MitoQ on the healing process in rabbit and rat models. The influence of MitoQ incorporation on the physico-chemical properties of the membranes is also determined.

## 2. Experimental

### 2.1. Materials and Animals

Chitosan of molar mass 100–300 kDa was obtained from ACROS Organics™ (Geel, Belgium). Hyaluronan (sodium salt) with a mean molar mass of 500.0 kDa was obtained from Contipro (Dolni Dobrouc, Czech Republic). NaOH, ethanol, acid citrate dextrose solution (ACD), calcium chloride solution, MTT 3-(4,5-dimethylthiazol-2-yl)-2,5-diphenyltetrazolium bromide, dimethyl sulfoxide (DMSO), phosphate-buffered saline solution (PBS), formaldehyde solution, trypane blue, diethyl ether, formalin, and haematoxylin and eosin were purchased from Sigma-Aldrich (St. Louis, MO, USA). MitoQ was a gift from the Medical Research Council Mitochondrial Biology Unit (Cambridge, United Kingdom).

Nine crossbred male rabbits HIL (2.5 ± 0.5 kg) from the Department of Toxicology and Breeding of Laboratory Animals at the Institute of Experimental Pharmacology and Toxicology in Dobra Voda, Slovakia were used. Fifteen adult female Wistar rats (230–250 g) were purchased from the Egyptian Company for Biological Products and Vaccines (VACSERA), Helwan, Cairo, Egypt. 

### 2.2. Composite Membrane Production

Membranes were prepared as follows: chitosan (0.5 g) was dissolved in 20 mL of aqueous acetic acid (2%, *v*/*v*). Hyaluronan (50 mg) was dissolved overnight (16 h) separately in 4 mL of water in accordance with Collins et al. (2013) [33]. Both solutions were then mixed together, and 1 mL of the aqueous stock MitoQ solution (1.47 mg/mL) was admixed into the homogeneous chitosan-hyaluronan solution. Next, 1 mL of glycerol was added into the chitosan-hyaluronan-MitoQ solution to function as a plasticizer. The multicomponent solution with a total volume of 25 mL was cast on a clean Petri dish (diameter = 7 cm) and the solvent was allowed to evaporate at room temperature over 48 h. The dry membrane, which was separated from the Petri dish, was rinsed for approx. 30 s in the aqueous NaOH solution (1 M). During this procedure, the traces of acetic acid, still adsorbed on the membrane, were removed thoroughly. The membrane was then washed for approx. 2 min in distilled water. Finally, the wet membrane was spread out and attached to a clean glass support with clamps, and allowed to dry for 24 h at room temperature. Prepared membranes were held between two polyethylene sheets and stored in a refrigerator (4 °C). The following membranes types were prepared: one without MitoQ (Ch/HA) and the other one containing MitoQ (Ch/HA/MitoQ). Before applying them to the rat skin, membranes were sterilized by spraying them with 70% aqueous ethanol, and dried under a UV lamp for 20 min.

### 2.3. Membrane Characterisation

Measurements of a static water contact angle were performed at room temperature using advanced 500-F1 model goniometer in a sessile drop configuration (using ultrapure water as the liquid), coupled with a video camera and image analysis software. At least ten droplet images were obtained for each membrane surface.

Approval was received from all volunteers before the use of their blood for assessment of compatibility and cytotoxicity. The human blood was used according to the Research Ethical Committee (REC) which was published by the National Health and Medical Research Council policies and the recommendations of Ministry of Health and Population, High Committee of Medical Specialties, Egypt. This current research was granted permission by City of Scientific Research and Technological Application (SRAT-City).

The membrane surface roughness was measured using a surface roughness tester (SJ-201P, Mitutoyo Corp., Kanagawa, Japan). Membranes were fixed onto a glass slide with double-sided tape. The dimensions of samples were 25 mm × 25 mm. All recorded results are the average of triplicate measurements.

Scanning electron microscopy equipped with energy dispersive spectroscopy (EDS) (JEOL Jsm 6360LA, Jeol Ltd., Tokyo, Japan) was utilized to analyse samples which were fixed on a specimen mount using carbon paste. All samples were coated with a thin layer of gold to eliminate charging effects. EDS was used to identify MitoQ in the final membranes through the identification of phosphorus atoms. Mechanical properties were measured according to ASTM D-882 using a universal testing machine (AG-1S Shimadzu, Schimadzu Corp., Tokyo, Japan). Membrane thickness was obtained with an electronic digital micrometer (Shenzhen Pride Instrument Inc., Guangdong, China).

### 2.4. Evaluation of Hemocompatibility of Membranes

Hemolysis tests were performed as described in American Society for Testing and Materials (ASTM) (ASTM F 756-00, 2000) (US Pharmacopeia XXIII, 1994). Anticoagulated blood was used for this purpose. This sample was prepared by adding 1 mL of anticoagulant acid citrate dextrose solution (ACD) to 9 mL of fresh human blood. Before performing the tests, the membranes (1 cm^2^) were placed in polypropylene test tubes, and 7 mL of PBS (pH 7.0) were added. After 72 h of incubation at 37 °C, PBS was removed, and 1 mL of ACD blood was added to each sample and maintained at 37 °C for 3 h. Positive and negative controls were prepared by adding the same amount of ACD blood to 7 mL of water and PBS, respectively. Each tube was gently inverted twice each for 30 min to maintain contact of the blood with the material. After incubation, each fluid was transferred to a suitable tube and centrifuged at 2000 rpm for 15 min. The hemoglobin released by hemolysis was measured by the optical densities (OD) of the supernatants at 540 nm using a spectrophotometer (Pharmacia Biotech Ultrospec 2000, Lab Wrench, Midland, ON, Canada).

The percentage of hemolysis was calculated as follows:

Hemolysis (%) = [(OD_sample_ − OD_negative control_)/(OD_positive control_ − OD_negative control_)] × 100 (1)

According to ASTM F 756-00 (2000), materials can be classified into three different categories based on their hemolytic index (%): (i) materials with percentages of hemolysis over 5% are considered hemolytic; while the ones with (ii) a hemolytic index between 5% and 2% are classified as slightly hemolytic. Finally, when the material presents (iii) a hemolysis percentage below 2%, it is considered as a non-hemolytic material.

### 2.5. Thrombogenicity of Membranes

Evaluation of thrombus formation on polymeric surfaces was carried out using a gravimetric method [34]. Anticoagulated fresh human blood was used for this purpose, as mentioned above. Before performing the tests, samples were immersed in PBS at a constant temperature of 37 °C. After 48 h of incubation, PBS was removed and ACD blood was put in contact with the surface of the tested membranes and also to the empty Petri dish, which acted as a negative control. Blood clotting tests were initiated by adding 20 µL of a 10 M calcium chloride solution and were stopped after 45 min by the addition of 5 mL of water. Resultant clots were fixed with 5 mL of a 36% formaldehyde solution and were then dried with a tissue paper and finally weighed. Each test was carried out in triplicate and mean values were calculated.

### 2.6. Determination of Toxicity against Human Peripheral Blood Mononuclear (PBMC) Cells 

The cytotoxic effect of different compound concentrations on normal human cell growth was determined using an MTT assay. Peripheral blood mononuclear cells (PBMC) were isolated using the Ficoll-Hypaque density gradient centrifugation method. Fresh heparinized blood was mixed with an equal volume of PBS, slowly layered over an equal volume of the Ficoll-Hypaque solution (density = 1.077 g/mL) and centrifuged for 30 min at 2000 rpm. The PBMC at the buffy layer was collected, resuspended in PBS, and centrifuged for 5 min at 1650 rpm. Cells were resuspended in RPMI 1640 containing 10% FBS (GIBCO, Waltham, MA, USA), counted, and viability was determined by staining with 0.5% trypan blue and counting on a hemocytometer (manufacturer, city, state, country).

Mononuclear cells (1 × 10^6^ cells/mL) were resuspended gently in RPMI medium (Lonza, Morristown, NJ, USA) containing 10% FBS and the cell count was adjusted to 1 × 10^5^ cells per 1 mL of the selected culture medium. The 1 × 10^5^ mononuclear cells were seeded in each well of the 96-well microtitre plate. Cells were treated with and without membranes of varying weights (4 and 5 mg) and wells containing only a complete medium were used as controls. After 72 h of incubation in a 5% CO_2_ incubator (Sanyo Electric Biomedical Co., Ltd., Osaka, Japan), 20 µL of MTT solution (5 mg/mL in PBS, pH 7) was added to each well and incubated at 37 °C for 4 h. The MTT solution was removed after centrifugation at 2000 rpm for 10 min and the insoluble blue formazan crystals trapped in cells were solubilized with 150 µL of 100% DMSO at 37 °C for 10 min. The absorbance of each well was measured with a microplate reader at 570 nm.

### 2.7. Ischemic Wound Healing in Rabbits 

Experiments were approved by the ethical committee of the Institute of Experimental Pharmacology and Toxicology in Bratislava, Slovakia (SK P 23011), followed by the State Veterinary and Food Administration in Bratislava, Slovakia (2267/16-221). Ischemic wounds on rabbits’ ears were performed according to DiPietro’s method [35]. Inside of each rabbit’s ear, two lacerations with a size of ca. 1 cm × 1 cm and a complete removal of skin tissue were performed. Rabbits were divided into three groups of three animals: first group—control (wound was covered with bandage); second group—animals treated with Ch/HA membrane; and third group—animals treated with Ch/HA/MitoQ membrane. After operation animals had a standard post-operational care. Animals were administered analgesics during the study. Rabbits were maintained individually in cages with an area of 4200 cm^2^ in daily 12 h light-dark cycles.

Animal wounds were covered with dehydrated membranes immediately after primary treatment of wounds. Each membrane was moisturized in saline and disinfected with 80% ethanol. During 15 day-experiment membranes were replaced with new ones after 3, 6, 9, and 12 days. Wounds in control animals were only washed with saline and in treated animals membranes were fastened to wounds with standard plasters. Wounded ears of all animals were bandaged. During this process animals were under anaesthesia. 

The efficacy of Ch/HA and Ch/HA/MitoQ membranes on healing of wounds was evaluated on the 3rd, 6th, 9th, 12th, and 15th day of the experiment through measuring the area of wounds. To statistically evaluate the percentage of healing in injured ischemic tissue, we used an ANOVA test. Results are shown as the average and standard deviation for each group of animals. Macroscopic morphology was evaluated from photographs.

### 2.8. Wound Healing in Rats 

The animal study was performed following the guidelines stipulated by the local committee of the Egyptian Academy of Scientific Research and Technology (ASRT) based on the protocols and instructions of the National Institutes of Health (Institute of Laboratory Animal Resources 1996) in the Faculty of Medicine, Alexandria University, Egypt. Rats were divided randomly into three groups with seven animals in each (five animals were analysed and two animals were sacrificed for histological study): group 1 where the wound was dressed directly with Ch/HA membrane followed by gauze and plaster; group 2 where the wound was dressed directly with Ch/HA/MitoQ membrane followed by gauze and plaster; and group 3 was the negative control, where the wound was covered with commercial cotton gauze supplied by El-Gomhouria Co. Cairo, Egypt and commercial plaster. After being anesthetized, their dorsal fur was shaved with an electric razor. The dorsum of rats was disinfected with 70% ethanol and wounds were created (1.5 cm × 1.5 cm). After being dressed with different membranes, which were previously sterilized in 70% ethanol and by UV lamp for 20–30 min, plasters were put on the dorsum and the whole abdomen of the rats. Bandages were used for fixing the dressing materials. Rats were kept separately in disinfected polypropylene cages and maintained for three weeks at 25 °C, as well as subjected to natural light for 12 h and dark for 12 h. The bandages were changed with a new membrane every three days until day 10. After 10 days of experiments, the bandages were changed only with gauze and plaster without adding any new membranes in the three groups. Wounds were cleaned with PBS after each membrane change. The healing wounds were observed and photographed using a digital camera on the 0th, 7th, 14th, and 21st day after surgery. With, the size of the healing wound determined and analysed using Image J software. The percentage of wound healing was estimated by using the following formula:

Wound healing (%) = 1 − (wound area day/wound area day 0) × 100(2)

All membranes were kept in a humidity chamber (37 °C and humidity 50%) for at least 6 h before being applied to the wound.

### 2.9. Histological Examination of Wounds

Rats were sacrificed after the 7^th^, 14^th^, and 21^st^ day, rabbits were sacrificed after the 15th day, and the wound areas, as well as the surrounding skin, were harvested for histological examinations. The tissues were immediately fixed in 4% formalin, then treated with conventional ethanol gradient dehydration and embedded in paraffin blocks. The tissues were sectioned into 6 µm thickness and stained with haematoxylin and eosin. Samples were subsequently observed under an optical microscope (Olympus, Tokyo, Japan).

## 3. Results and Discussion

### 3.1. Identification of MitoQ within Membranes

**Figure 1:**
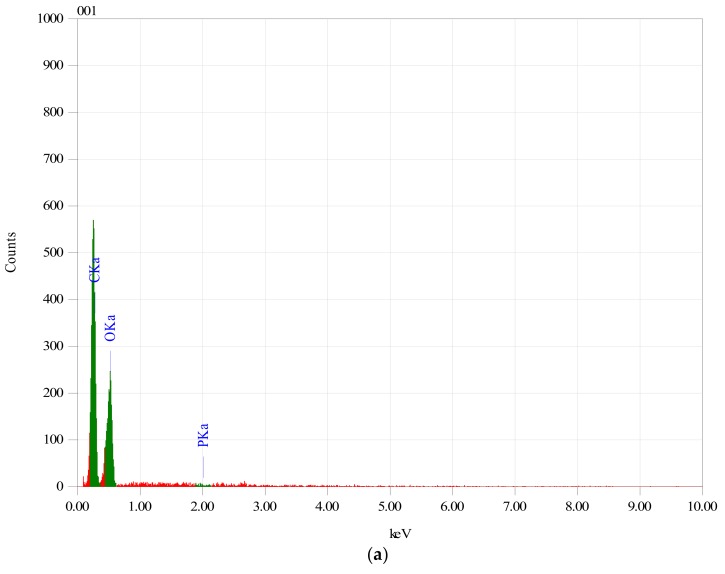

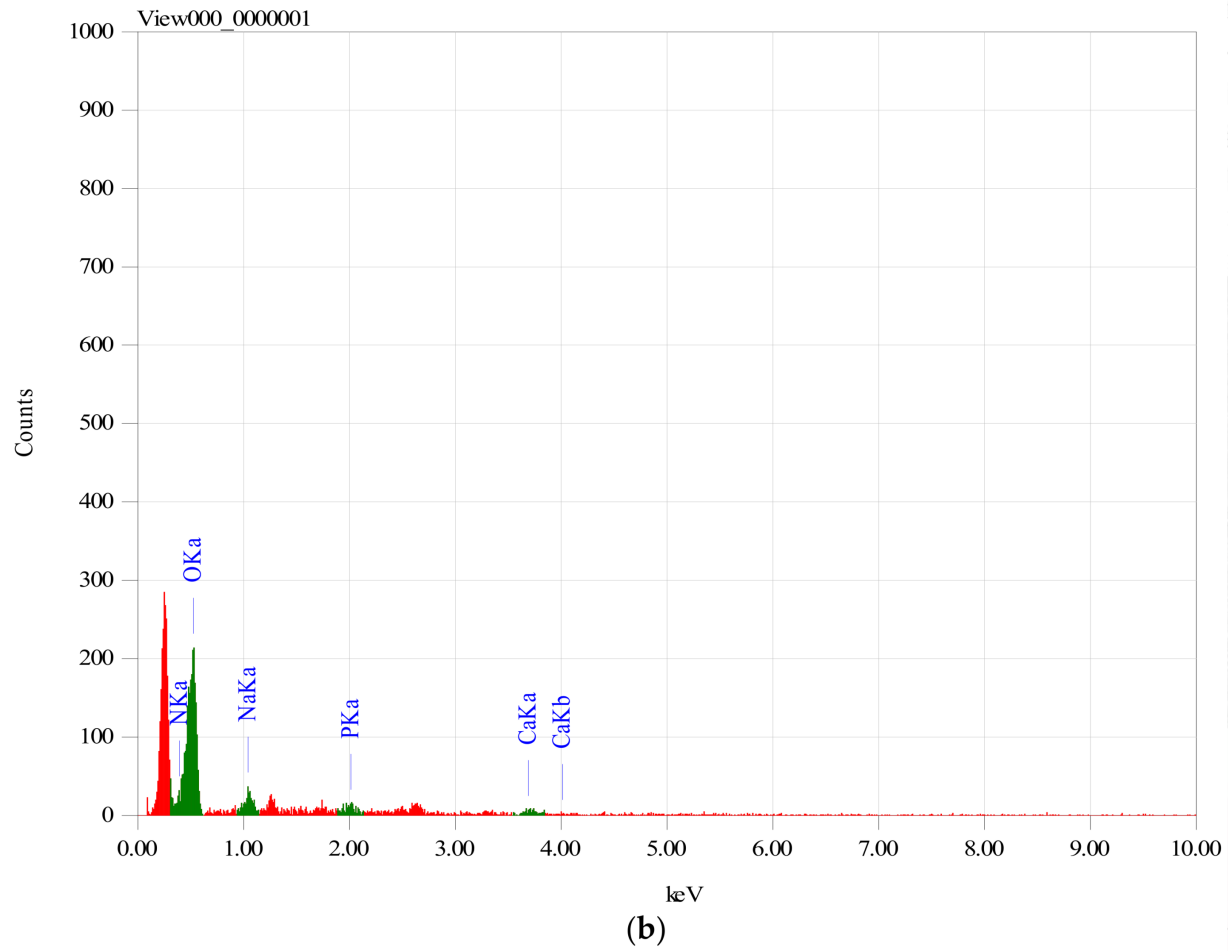
EDX spectrum of (**a**) Ch/HA and (**b**) Ch/HA/MitoQ membranes.

In order to establish if the MitoQ was successfully incorporated within the Ch/HA membranes, membranes which were subjected to the MitoQ loading regime were studied by EDS. Figure 1 clearly shows the EDS spectrum of membranes with and without MitoQ. Membranes containing MitoQ displayed phosphorous (~2.64%) which confirmed that MitoQ was successfully incorporated in those membranes. The absence of phosphorus in the control membranes confirmed the result. 

### 3.2. Membrane Characterization

The contact angles of Ch/HA and Ch/HA/MitoQ membranes were recorded as 70.1 ± 0.48° and 77.4 ± 0.47°, respectively. Ch/HA membranes became more hydrophilic than chitosan membranes (contact angle of pure chitosan film was 89 ± 0.6°). An increase in membrane hydrophobicity is associated with MitoQ loading. Additionally, the roughness of membranes increased from 1.1 ± 0.12 to 1.23 ± 0.31 µm, as a result of MitoQ addition.

One of the most important conditions for enhancing the wound healing process is the existence of a moist environment over the wounded site. Dressing agents that provide a moist burn or wound environment boost the healing process via augmenting cell migration to the injured area and these cells aid tissue regeneration. Additionally, the hydrophobicity and roughness of the membranes prevent water absorption from the wound aiding the overall healing process.

There is also evidence of a slight increase in hemolysis (2.2 ± 0.14% to 2.3 ± 0.28%) by MitoQ containing membranes. With respect to thrombogenicity, blood clots were 0.821 ± 0.021 and 0.927 ± 0.012 g for Ch/HA and Ch/HA/MitoQ, respectively. The clots associated with the membranes were larger than those associated with the negative control (blood without membranes) which were 0.729 ± 0.021 g. For this reason the membranes can be classified as thrombogenic [34].

Cytotoxicity of Ch/HA and Ch/HA/MitoQ on normal human cell growth was measured and listed in Table 1. Cell viability reduced as a function of membrane weight and improved with incorporation of MitoQ into the membranes. 

**Figure 2:**
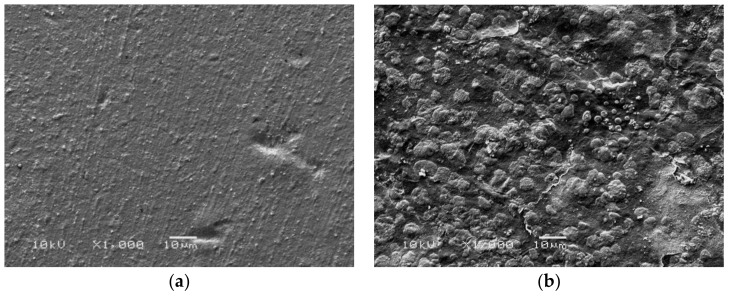
SEM images of (**a**)Ch/HA and (**b**)Ch/HA/MitoQ membranes.

Figure 2 shows the morphology of Ch/HA and Ch/HA/MitoQ membranes. Incorporation of MitoQ inside membranes shows increased surface roughness. This is explained by MitoQ-induced crystal structure distortion and elimination of intermolecular bonds. The increased surface roughness of membranes will help achieve a favourable interaction between the membranes and biological tissues.

Mechanical properties of membranes with and without MitoQ are shown in Table 2. The tensile strength of Ch/HA membranes is attributed to the intermolecular hydrogen bonds between hydroxyl and amine groups in chitosan and the hyaluronan backbone and polyelectrolyte interaction between carboxylic and amine groups. Addition of MitoQ demonstrates a reduction in mechanical properties which may be attributed to a plasticization effect; a similar trend was shown in caprolactones upon additive addition [36,37] or the interruption of chain-chain intermolecular bonding. Overall, the mechanical measurements show that MitoQ molecules slightly disturb the membrane formation which occurs through polyelectrolyte ion-pair interactions as the resistance at break is slightly reduced.

### 3.3. Reepithelization of Ischemic Wounds in Rabbits

**Figure 3:**
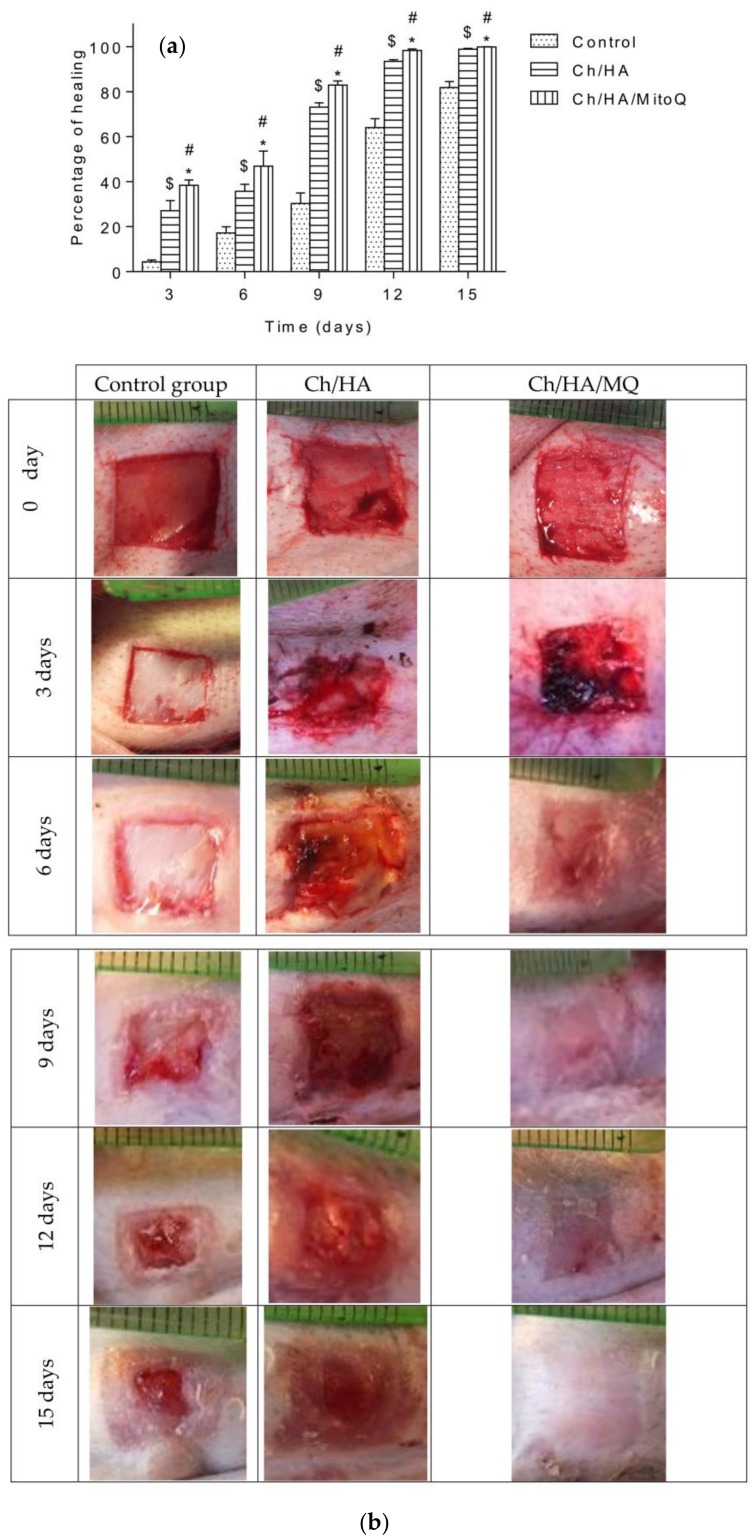
Profiles of the wound closures in rabbits, when the wound was not treated (control), the wound treated with the Ch/HA membrane and the Ch/HA/MitoQ membrane. The asterisk (*) indicates a significant difference at *p* ≤ 0.05 between the control and Ch/HA/MitoQ. The mark (^#^) indicates a significant difference between the Ch/HA and Ch/HA/MitoQ membranes at *p* ≤ 0.05. The mark (^$^) indicates a significant difference between the control and the Ch/HA membrane at *p* ≤ 0.05, *n* = 6 (a, b).

The prepared membranes were applied on two animal models: rats and rabbits. Two animal models were used, as rats are a model for healthy animals and rabbits with ear ischemia are a model for animals with disease. As seen in Figure 3 a, b results of measuring areas of wounds after three days showed only mild shrinking of the wound area in untreated control animals. On the contrary, in rabbits treated with Ch/HA and Ch/HA/MitoQ membrane faster healing was observed. On the 6th day a significantly faster re-epithelisation of treated skin wounds was seen compared to the control group. Re-epithelisation of the tissue in the control group of animals was observed on the 9th day of the study. After 12 days the process of wound healing accelerated in all groups, especially in treated ones. In untreated animals the percentage of shrinking wounds after 15 days reached 81% while, in treated animals, the percentage of wound healing was over 90% with some wounds completely healed or re-epithelised. It can be concluded that the fastest re-epithelisation was observed in wounds covered with Ch/HA/MitoQ composite membranes. Ch/HA membranes were shown to be less effective in healing rabbits’ wounds.

In the control group maturating granular tissue with the prevalence of leukocytes, lower amount of histiocytes, perpendicular distribution of fibroblasts, and hyperemic capillaries with perivascular bleeding can be observed, with corresponding images of wound contraction in rabbits at the 0^th^, 3^rd^, 6^th^, 9^th^, 12^th^, and 15^th^ day after injury in the control group and groups covered with Ch/HA and Ch/HA/MitoQ membranes.

In the skin wound treated with the Ch/HA membrane we can see maturating granular tissue and it is expected that this would be composed predominantly of macrophages, plasmocytes, leukocytes, fibroblasts, and myxoid changes of the stroma due to the presence of acid mucopolysaccharides.

**Figure 4:**
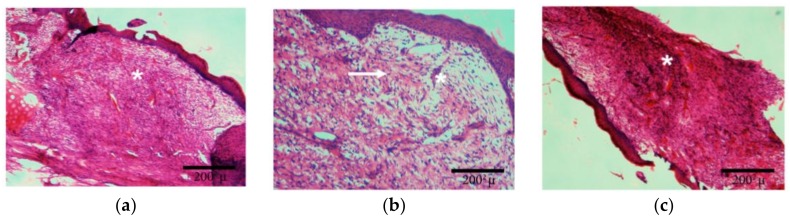
Histology of skin wounds in rabbits after 15 days of treatment. (**a**)Control: star—vascular granular tissue; (**b**)Ch/HA membrane: arrow—likely site of activated fibroblasts with formation of collagen fibres (it is acknowledged that a Masson trichrome or immunostaining is required to accurately identify these entities), star—newly-formed veins; (**c**)Ch/HA/MitoQ membrane: star—nonspecific granular tissue in the initial stage with enhanced cellularity.

As shown in Figure 4, the wound treated with the Ch/HA/MitoQ membrane is a healed skin defect that is likely composed of maturated granular tissue with histiocytes, and small amounts of leukocytes, fibroblasts, and myxoid changes in the stroma may be due to the accumulation of acid mucopolysaccharides.

### 3.4. Experiments In Vivo in Rats

**Figure 5:**
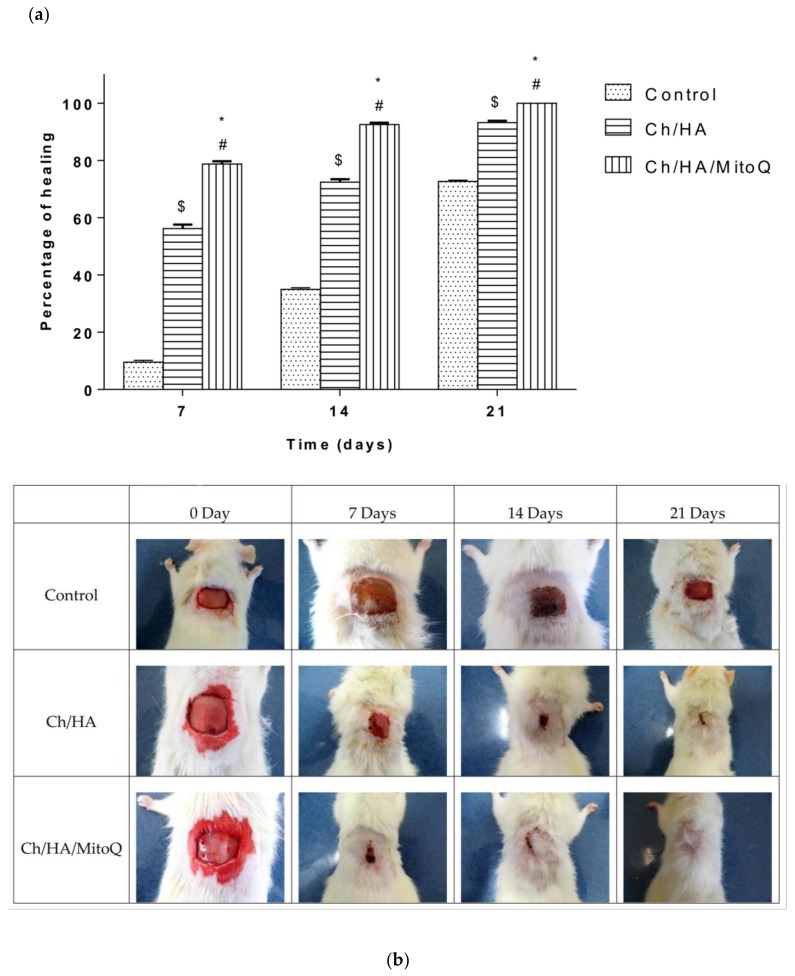
Profiles of the wound closures in rats, when the wound was not treated (control), the wound treated with the Ch/HA membrane, and the Ch/HA/MitoQ membrane; and the asterisk (*) indicates a significant difference at *p* ≤ 0.05 between the control and Ch/HA/MitoQ). The mark (^#^) indicates a significant difference between the Ch/HA and Ch/HA/MitoQ membranes at *p* ≤ 0.05. The mark (^$^) indicates a significant difference between the control and the Ch/HA membrane at *p* ≤ 0.05, *n* = 5 (a, b).

Wound closure percent was calculated and is illustrated in Figure 5a. Photographs of the full layer wounds treated with Ch/HA and Ch/HA/MitoQ composite membranes and control (gauze) dressings during 0–21 days after injury are shown in Figure 5b. Both Ch/HA and Ch/HA/MitoQ dressings kept the wounds moist. At the day of surgery, no noticeable variation in a wound appearance was noted for all groups. On the 7th day it is clear both Ch/HA and Ch/HA/MitoQ dressing promoted the fastest healing. On the 14th day, wounds treated with the Ch/HA/MitoQ were almost healed. On the 21st day, the damaged skin was entirely healed for the dressing with Ch/HA/MitoQ composite membranes. Visual observations indicate that the Ch/HA membranes act as efficient wound healers and their performance can be further enhanced through the incorporation of mitochondrially-targeted antioxidant—MitoQ. 

### 3.5. Histological Observation

**Figure 6:**
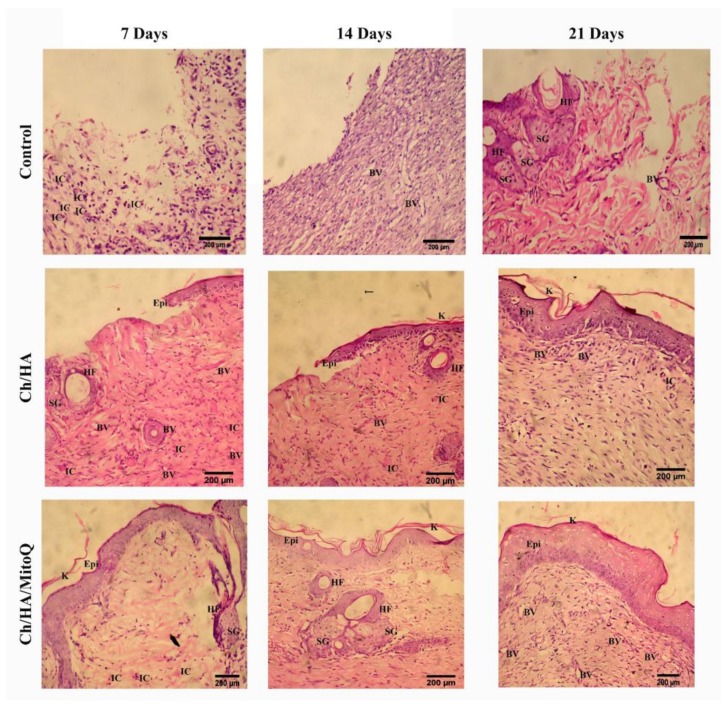
Histology of wounds treated with control (gauze), Ch/HA, and Ch/HA/MitoQ membranes at the 7^th^, 14^th^, and 21^st^ day after the injury. Epi: Epidermis; K: keratinous layers; IC: possible site of inflammatory cells; BV: blood vessels; HF: hair follicles; SG: sebaceous glands.

Figure 6 shows the histological analysis of injured tissue treated with Ch/HA and Ch/HA/MitoQ membranes compared with the untreated wound (control) at different times after injury. Recovery of tissue was clearly observed with CH/HA/MitoQ membranes at all time intervals. Positive control (Ch/HA) showed significant healing comparing to the negative control (gauze). The control group had a significantly higher number of inflammatory cells than that one treated with membranes of any type, i.e., with or without MitoQ. Efficient healing was verified by the presence of hair follicles, and matured fibrous tissues for membranes containing MitoQ. 

Traditional treatment of acute wounds using gauze only (as in the control group here) exhibited incomplete healing during the study period. Applying Ch/HA membranes to the wound demonstrated an improvement in wound healing compared to the control and this is attributed to faster migration of epithelium cells to replace dead cells. For composite membranes (i.e., Ch/HA/MitoQ) the histology of rat skin preparations showed significant development in the initial healing step when compared to both the negative control (gauze) and the positive control (Ch/HA membrane). This observation can be attributed to the MitoQ control of wound inflammation during the initial healing phase.

## 4. Conclusions

These carbohydrate polymers (Ch and high-molar-mass HA) are known to have a beneficial effect on the healing process and induction of homeostasis in organisms. Here, it has been demonstrated that the Ch/HA/MitoQ composite membrane, when subjected to physical-chemical and mechanical tests, displayed different properties compared to the Ch/HA membrane only. When demonstrated on injured rabbit and rat skin the membranes were remarkably efficient, and accelerated the healing process mainly through an inflammation suppression mechanism. The results reported here of in vivo experiments show the potential of using MitoQ—an MTA substance—for the treatment of skin tissue wounds. 

## Figures and Tables

**Table 1:** Cytotoxicity studies of Ch/HA and Ch/HA/MitoQ membranes.

	Cell Viability [%]	
Sample Weight (mg)	Ch/HA	Ch/HA/MitoQ
5	80.62 ± 2.3	86.69 ± 4.4
4	85.16 ± 2.8	98.69 ± 5.7

**Table 2:** Mechanical properties of Ch/HA and Ch/HA/MitoQ membranes.

	Max Stress σ_max_ (N/mm^2^)	Max Strain λ_max_ %	Energy–Max (J)
Ch/HA	34.06 ± 1.9 *n* = 3	4.9 ± 1.7 *n* = 3	0.04 ± 0.01 *n* = 3
Ch/HA/MitoQ	22.0 ± 1.6 *n* = 3	5.04 ± 1.5 *n* = 3	0.01 ± 0.01 *n* = 3
